# Non-functioning pituitary adenomas: indications for pituitary surgery and post-surgical management

**DOI:** 10.1007/s11102-019-00960-0

**Published:** 2019-04-22

**Authors:** Daniela Esposito, Daniel S. Olsson, Oskar Ragnarsson, Michael Buchfelder, Thomas Skoglund, Gudmundur Johannsson

**Affiliations:** 1 Department of Endocrinology, Institute of Medicine, Sahlgrenska University Hospital, Sahlgrenska Academy, University of Gothenburg, SE-413 45 Gothenburg, Sweden; 2 Department of Neurosurgery, University of Erlangen-Nürnberg, Schwabachanlage 6, 91054 Erlange, Germany; 3 Department of Neurosurgery, Sahlgrenska University Hospital, SE-413 45 Gothenburg, Sweden

**Keywords:** Pituitary adenomas, Hypopituitarism, Endocrine care, Pituitary surgery, Surgical outcome

## Abstract

**Purpose:**

Non-functioning pituitary adenomas (NFPAs) are associated with impaired well-being, increased comorbidities, and reduced long-term survival. Data on optimal management of NFPAs around surgical treatment are scarce, and postoperative treatment and follow-up strategies have not been evaluated in prospective trials. Here, we review the preoperative, perioperative, and early postoperative management of patients with NFPAs.

**Methods:**

We searched Medline and the Cochrane Library for articles published in English with the following items “Pituitary neoplasms AND Surgery” and “Surgery AND Hypopituitarism”. Studies containing detailed analyses of the management of NFPAs in adult patients, including pituitary surgery, endocrine care, imaging, ophthalmologic assessment and long-term outcome were reviewed.

**Results:**

Treatment options for NFPAs include active surveillance, surgical resection, and radiotherapy. Pituitary surgery is currently recommended as first-line treatment in patients with visual impairment due to adenomas compressing the optic nerves or chiasma. Radiotherapy is reserved for large tumor remnants or tumor recurrence following one or more surgical attempts. There is no consensus of optimal pre-, peri-, and postoperative management such as timing, frequency, and duration of endocrine, radiologic, and ophthalmologic assessments as well as management of smaller tumor remnants or tumor recurrence.

**Conclusions:**

In clinical practice, there is a great variation in the treatment and follow-up of patients with NFPAs. We have, based on available data, suggested an optimal management strategy for patients with NFPAs in relation to pituitary surgery. Prospective trials oriented at drawing up strategies for the management of NFPAs are needed.

## Introduction

Non-functioning pituitary adenomas (NFPAs) are benign tumors arising from the adenohypophyseal cells characterized by the absence of clinical evidence of hormonal hypersecretion. According to recent epidemiological data, the estimated prevalence of NFPAs is 7–41.3 cases/1,00,000 and the annual incidence is 0.65–2.34 cases/1,00,000 [1–3]. The incidence of NFPAs has increased over time, most probably due to an increasing number of incidentally discovered adenomas on brain imaging performed for unrelated reasons (pituitary incidentalomas) [4].

According to the 4^th^ edition of the World Health Organization classification of endocrine tumours (WHO 2017), NFPAs can be categorized into eight subtypes: silent gonadotroph, corticotroph, somatotroph, thyrotroph, lactotroph, plurihormonal Pit-1, null-cell, and double/triple NFPAs. This classification takes into account the immunohistochemical expression of pituitary hormones and pituitary-specific transcriptional factors. However, the correlation between histopathological factors and clinical behavior of NFPAs remains unclear and reliable immunohistochemical predictors of aggressiveness in NFPAs are still lacking [5, 6].

Clinical features due to NFPAs vary greatly. Some patients are asymptomatic while others have signs and symptoms due to mass effects on surrounding structures, causing headache, visual defects, and/or hypopituitarism [2, 7].

Careful clinical examination as well as endocrine, radiological, and ophthalmological assessment determine the best treatment strategy. However, new molecular markers are needed in order to further personalize and optimize treatment approaches [8].

Although NFPAs are histologically benign tumors, there are data showing that patients suffering from NFPAs have increased comorbidities and excess mortality [3, 9, 10]. Tumor treatment and follow-up strategies lack evidence from randomized studies and great variation in clinical practice has been reported [11].

Herein, we review the endocrine and surgical care of patients with symptomatic NFPAs with a focus on preoperative, perioperative, and postoperative management, and put this into context with the long-term outcome of these patients.

### Clinical presentation

Patients with symptomatic NFPAs commonly present with symptoms related to the mass effect on surrounding structures, including headache, visual defects, and hypopituitarism [7].

Headache is reported to be present in 16–70% of patients with pituitary adenomas [12–15]. Tumor enlargement leads to stretching of the diaphragm of the sella with activation of pain fibers within the dura mater, resulting in headache, mainly localized in the frontal and occipital regions [16, 17].

Patients with large pituitary adenomas can also present with visual impairment, classically bitemporal visual defects related to mid-chiasmal compression [18, 19]. In a recent meta-analysis including a 35-case series, the frequency of visual field deficits at diagnosis ranged between 28 and 100% [20]. NFPAs may also grow asymmetrically, leading to different patterns of visual field defects [19]. Diplopia is rare, but when present, is caused by compression of the cavernous sinus [20]. Its presence should raise a suspicion of another cause rather than a pituitary adenoma.

The mechanical compression of normal pituitary cells, pituitary stalk, and portal vessels may lead to hormone deficiencies, hyperprolactinemia, and, rarely, diabetes insipidus (DI). The prevalence of hypopituitarism at diagnosis ranges between 37 and 85%, depending on the tests and criteria used [21–23].

Patients with NFPAs may rarely present with pituitary apoplexy, which is a rare endocrine emergency caused by an acute infarction or hemorrhage in the tumor [24, 25]. Common clinical features include sudden severe headache, visual loss, nausea, vomiting, impaired consciousness, symptoms of meningeal irritation, and acute endocrine dysfunction [26]. The optimal management of this acute and potentially life-threatening condition is challenging; the role and timing of neurosurgical decompression is still controversial [27].

### Preoperative evaluation

#### Endocrine assessment

According to clinical guidelines, all patients with pituitary macroadenomas and larger microadenomas (6–9 mm), with or without symptoms, should undergo laboratory assessment in order to detect hormonal hypersecretion or hypopituitarism [28, 29] (Table 1).

**Table 1:** Summary of the pre-, peri- and postoperative management of NFPAs

Preoperative management	
Endocrine assessment	
∙ Rule out a hormone-producing adenoma clinically and biochemically	
∙ HPA axis	- Morning serum cortisol; dynamic testing if needed - Introduce GC replacement if SAI is confirmed
∙ Thyroid	- Serum TSH and free T4 - Introduce L-thyroxine in severe CH
∙ HPG axis	- Evaluate hypogonadism clinically and biochemically - Sex hormone replacement is usually not indicated preoperatively
∙ Somatotropic axis	- Diagnosis and/or treatment for GHD is not recommended preoperatively
Radiological assessment	
∙ MRI evaluating the relationship to the chiasma and optic nerve, and grading of extrasellar extension using the Knosp scale	
Ophthalmologic assessment	
- Visual field, visual acuity, and eye movement	
Perioperative and early postoperative management	
∙ GC therapy	- Administrate stress doses of GCs in patients with confirmed and suspicion of SAI - Monitor morning serum cortisol regularly in patients without SAI who do not receive GCs perioperatively - Introduce GCs if cortisol deficiency is detected
∙ Fluid balance	- Monitor urine volume and serum sodium regularly to detect hyponatremia and/or DI
Postoperative management	
Endocrine assessment	
∙ HPA axis	- Re-evaluation of HPA axis with morning serum cortisol and a dynamic testing, if needed, after 6–12 weeks
∙ Thyroid	- Morning serum TSH and free T4 - In case of CH, introduce L-thyroxine only after HPA axis has been assessed and cortisol deficiency corrected
∙ HPG axis	- Clinical and biochemical evaluation of hypogonadism - Introduce sex hormone replacement in pre-menopausal women, if needed - Introduce testosterone replacement in men, if needed
∙ Somatotropic axis	- Assess GHD after 6–12 months and only after any other hormone deficiency is adequately replaced - Introduce GH replacement therapy if GHD is confirmed
Radiological assessment	
∙ Perform the first MRI 3–6 months following surgery ∙ Subsequent follow-up is individualized based on MRI findings and histopathological diagnosis	
Ophthalmologic assessment	
∙ First examination within 3 months ∙ Patients with postoperative visual defects need further follow-up	

*CH* central hypothyroidism, *DI* diabetes insipidus, *GC* glucocorticoid, *GH* growth hormone, *GHD* growth hormone deficiency, *HPA* hypothalamus–pituitary–adrenal, *HPG* hypothalamus-pituitary–gonadal, *MRI* magnetic resonance imaging, *NFPA* non-functioning pituitary adenoma, *SAI* secondary adrenal insufficiency, *TSH* thyroid-stimulating hormone

Growth hormone (GH) deficiency and hypogonadism are the most commonly found deficits followed by central hypothyroidism and secondary adrenal insufficiency [7, 30]. Panhypopituitarism is present at diagnosis in 6–29% of patients [31]. DI is a rare finding at diagnosis of NFPAs. Therefore, in patients presenting with DI and a pituitary mass, other tumors than NFPAs should be considered [32–35].

At diagnosis, 25–65% of patients with NFPAs present with hyperprolactinemia caused by pituitary stalk compression [12, 21, 30]. It is important to distinguish between a prolactinoma and a NFPA since treatment strategies for these two conditions differ, i.e. dopamine agonist therapy being the treatment of choice for prolactinomas [36]. In a retrospective analysis of 117 patients with prolactinomas and NFPAs, it was found that NFPA patients most often had a prolactin (PRL) level < 100 ng/mL (~ 2000 IU/L) whereas levels > 250 ng/mL (~ 5000 IU/L) were exclusively seen in patients with prolactinomas [37]. There is a large grey zone between these two thresholds where individual clinical judgement needs to be used when deciding the primary choice of treatment.

#### Radiological assessment

Magnetic resonance imaging (MRI) with and without gadolinium contrast is the gold standard for morphological assessment of pituitary adenomas [38]. NFPAs usually appear hypointense or isointense on T1-weighted images. After contrast administration, pituitary adenomas exhibit delayed enhancement, appearing hypointense in relation to the pituitary gland, which has an earlier and more intense enhancement. In the case of atypical radiological findings, other diseases should be considered, e.g. hypophysitis, meningioma, granulomatous disorders, metastases [39]. MRI is crucial for staging and surgical planning since it shows, with high accuracy, the relationship of the adenoma to the chiasma and to the carotid arteries as well as the degree of invasion into the cavernous sinuses.

Based on size, pituitary adenomas can be classified into microadenomas (< 1 cm), macroadenomas (≥ 1 cm), and giant adenomas (≥ 4 cm). Another clinically and prognostically relevant radiological classification was introduced by Knosp and colleagues [40], which was revised in 2015 [41]. The classification consists of a grading system of parasellar adenoma extension, with grade 0 corresponding to an adenoma without any parasellar extension and grade 4 to the total encasement of the intracavernous carotid artery. The parasellar adenoma extension is considered to be a negative prognostic factor for surgical outcome [41].

#### Ophthalmologic assessment

A complete neuro-ophthalmologic evaluation, including visual field and acuity examination, is required in case of visual complaints or if the tumor abuts the optic chiasm or optic tract on MRI. Ophthalmologic assessment should also be performed in order to be able to judge the operative impact on any pre-operative abnormalities [42].

In patients with microadenomas or macroadenomas remote from the chiasma and cavernous sinus, neuro-ophthalmological assessment is not required [43]. In patients with NFPAs in contact with the optic chiasm, strict ophthalmologic surveillance should be performed in the case of conservative management. In these patients, the onset of new visual defects is a strong indication for surgery [11, 44].

### Indication for surgery and perioperative management

**Fig. 1:**
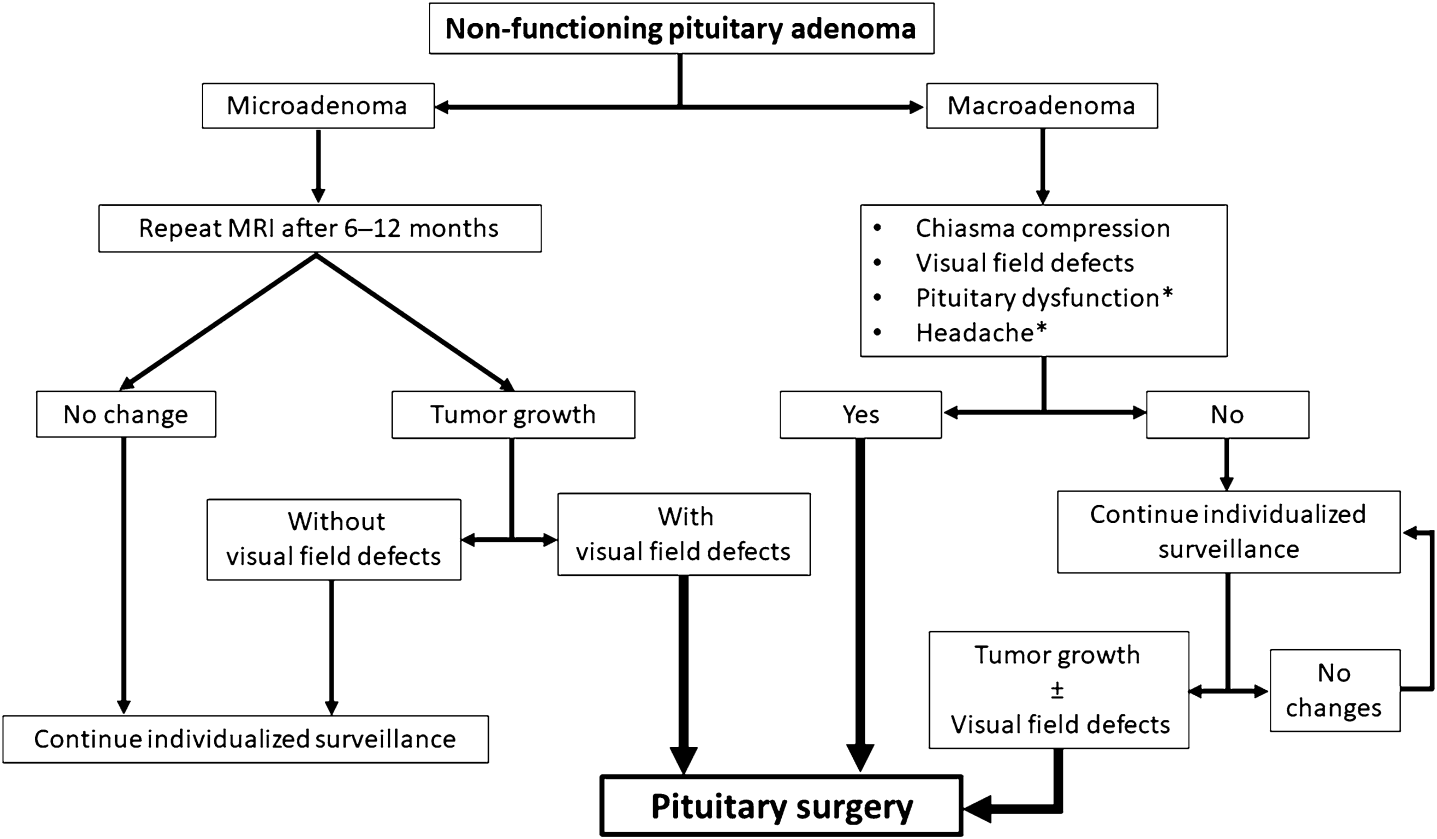
Indication for pituitary surgery in patients with non-functioning pituitary adenomas. Surgery is currently recommended in patients with adenomas abutting or compressing the chiasma with visual field deficits. In the absence of visual impairment, a conservative management may be considered. In these cases, an individualized surveillance including hormonal, radiologic, and ophthalmologic assessment is suggested. *Hypopituitarism and headache alone are not a strong indication for surgery because improvement in pituitary function and relief from headache cannot be guaranteed. Therefore, treatment decision should be individualized and based on clinical context and patient preference

Treatment options for NFPAs include active surveillance, surgical treatment, and radiotherapy. In patients with large NFPAs and visual impairment or other signs and symptoms related to tumor compression, transsphenoidal surgery is the recommended first-line treatment [28] (Fig. 1). Radiotherapy, as a primary therapy, is only considered in cases where surgery is contraindicated, such as in patients with other serious co-morbidities or in inoperable cases [45].

The goal of surgical treatment is to provide symptom relief, preserve the surrounding neural structures, and prevent deterioration of vision and pituitary function as well as to reverse any functional impact on visual nerves, chiasma, and the pituitary gland.

#### Symptomatic non-functioning pituitary adenoma

Surgery is the recommended treatment in patients with visual field deficits or other visual abnormalities, adenomas abutting or compressing the optic nerves or chiasm, and in patients with pituitary apoplexy with visual disturbances [28]. In the absence of visual impairment, the optimal treatment choice is still a matter of debate, especially in patients presenting with hypopituitarism, headache, or tumors close to the chiasma. Surgery may improve pituitary function in up to 30% of patients with pre-existing hypopituitarism [46], but the risk of new hormone deficiency following surgery is 2–15% [47, 48]. Therefore, hypopituitarism alone is not an indication for surgical treatment. Unremitting headache may be an indication for surgery even though relief cannot be guaranteed (Fig. 1).

#### Asymptomatic non-functioning pituitary adenomas

Surgical resection of non-functioning microadenomas is not indicated since tumor growth is rare (3–13%) with less than 5% growing > 1 cm during long-term follow-up [43, 49–51]. Management of non-functioning microadenomas is outside the scope of this review.

Management strategies of asymptomatic non-functioning macroadenomas vary greatly [52, 53]. The median rate of tumor enlargement in macroadenomas has been reported to be 0.6 mm/year [11]. Conservative management is recommended for macroadenomas not reaching the optic chiasm with regular surveillance of tumor status and endocrine function [18] (Fig. 1). However, treatment decisions should be individualized and based on age, pituitary function, and patient preference [52]. Surgery may be favored in younger patients given the higher lifetime probability of tumor growth and discouraged in older patients with comorbidities and risk of surgical complications [54].

Despite NFPAs usually have a slow growth rate, some may enlarge and become symptomatic. Biochemical evaluation for hypopituitarism should therefore be considered every 6–12 months during conservative management because remaining pituitary function may deteriorate by tumor enlargement [11, 28]. Radiological assessment by MRI should be repeated within 6–12 months after initial tumor detection; if no progression is detected, MRI can be performed less often [28]. The timing of visual field follow-up usually depends on the distance between the adenoma and the optic chiasm [54].

#### Perioperative endocrine care

Patients with confirmed secondary adrenal insufficiency should be adequately treated with glucocorticoid (GC) replacement therapy and stress GC doses should be administered during the perioperative period [55, 56] (Table 1). Perioperative GC therapy is also frequently used in patients with intact hypothalamus–pituitary–adrenal (HPA) function. The rationale is to cover these patients in case adrenal insufficiency develops during the surgical procedure [55, 56]. Cortisol response to major surgical stress has been shown to last for 48 h in healthy subjects [57]. Based on this, it has been suggested to discontinue GC therapy 48 h after surgery [55, 58]. However, in many centers, GC therapy is administered in tapering doses and then discontinued when proper re-evaluation of HPA has been performed [56].

Patients with preoperative overt central hypothyroidism should receive thyroxine replacement therapy before surgery. Patients with severe hypothyroidism have increased risk of surgical complications [59]. Therefore, in case of non-emergency surgery, it is suggested to wait until thyroxine replacement therapy has been initiated and optimized [56].

#### Surgical technique

The current standard technique for most NFPAs is endoscopy or microscopy assisted transsphenoidal surgery (TSS), while the transcranial approach is used for predominantly suprasellar tumors which lack significant intrasellar portions [60]. The endoscopic technique is to date widely used. However, from a global viewpoint, the majority of TSS is still performed microsurgically. Although the microscopic and endoscopic techniques have been available side by side for more than 20 years, there is still no convincing proof for superiority of one or the other. Thus, the controversial discussion of which visualization technique is associated with a higher rate of gross total resection and a lower risk of complications continues [61, 62].

Intraoperative MRI is being increasingly introduced into pituitary surgery. Intraoperative imaging shows the tumor status during the surgery, making it possible to continue surgical resection of a tumor remnant. Hypothetically, intraoperative MRI may improve surgical outcomes. However, the usefulness of the technology is still controversial, with some studies reporting a higher rate of gross total resection [63, 64] but others showing no difference [65].

#### Surgical outcomes and complications

Gross total resection is achieved in 60–73% of patients with NFPAs [61]. In a recent meta-analysis on NFPA patients, TSS was associated with 1% mortality [46]. Postoperative complications such as cerebrospinal fluid (CSF) leakage, fistula, meningitis, vascular injury, persistent DI, or new visual field defect occurred in ≤ 5% of patients [46]. Surgical complications are reported to be less frequent with higher-volume surgeons or hospitals [66]. The risk of CSF leakage is increased in patients with large adenomas with suprasellar extension, intraoperative CSF leakage, repeat TSS, and high body mass index [67, 68].

### Postoperative management

There is a lack of evidence on timing, frequency, and duration of postoperative endocrine, radiologic, and ophthalmologic assessments. However, recent reviews offer practical advice during the postoperative management of NFPAs [69, 70]. Most studies describe postoperative endocrine evaluation 4–8 weeks after the surgical procedure and others 2–6 months postoperatively.

In the early postoperative phase, patients should be carefully monitored for potential surgical complications, including sellar hematoma, CSF leakage, meningitis, hydrocephalus, and epistaxis. If neurological symptoms, significant rhinorrhea, or new visual impairments occur after surgery, an early postoperative computerized tomography or sellar MRI should be performed [71]. Potential endocrine complications include acute adrenal insufficiency and electrolyte abnormalities. Unrecognized secondary adrenal insufficiency in the postoperative period can result in adrenal crises and even death [72]. Morning cortisol levels, electrolytes, and urine production should be carefully monitored in the early postoperative period [73, 74] (Table 1).

#### Postoperative endocrine assessment

##### Transient syndrome of inappropriate antidiuretic hormone secretion (SIADH)

SIADH may occur within the first 3–7 days postoperatively, with an incidence ranging from 4 to 20% [75]. Transient SIADH is due to iatrogenic manipulation of the posterior pituitary gland resulting in excessive antidiuretic hormone (ADH) release [76, 77]. In rare cases, it may result in severe, life-threatening, acute hyponatremia [75].

Treatment strategies include fluid restriction, hypertonic saline administration, or vasopressin two receptor antagonist treatment [77]. It is important to avoid excessive administration of intravenous fluids in the postoperative period and prophylactic fluid restriction is recommended by some during the first 10 days after surgery in order to reduce SIADH frequency or minimize the degree of hyponatremia due to SIADH [75, 77, 78].

##### Diabetes insipidus

DI occurs in 18–31% of patients after pituitary surgery [77, 79]. Several factors are associated with the increased risk of postoperative DI, including male sex, young age, large pituitary mass, CSF leak, and administration of high perioperative glucocorticoid doses [77, 80]. In most patients, the disease is transient, being caused by the temporary dysfunction of ADH-secreting neurons. It usually occurs 24–48 h postoperatively and resolves when ADH-secreting cells recover their normal function [77].

Triphasic DI occurs in 3–4% of patients. The first phase is characterized by DI (usually 5–7 days) due to a partial or complete posterior pituitary dysfunction. The second phase is caused by an uncontrolled release of ADH leading to SIADH, which usually lasts 2–14 days. Finally, the last phase occurs if > 80–90% of the ADH-secreting cells have degenerated, which leads to permanent DI [77].

Postoperative DI should be suspected if polyuria (≥ 3 L per day) and polydipsia occur in combination with low urine osmolality. Serum hyperosmolality and hypernatremia strongly support the diagnosis of DI. In this clinical context, a water deprivation test is not needed [81, 82]. A urine osmolality < 300 mOsm/kg and subsequent positive response to ADH confirms the diagnosis of central DI [82].

In patients who are able to drink in response to thirst and when sodium levels remain within the normal range, no treatment is needed. In other cases, treatment with desmopressin may be required [83]. In treated patients, urine output and osmolality, as well as serum sodium levels, should be monitored regularly to avoid hyponatremia. Because postoperative DI can be transient, each dose of desmopressin should be administered after the recurrence of polyuria and thirst. This approach allows recognition of restored ADH secretion and transient DI in the early and late postoperative phases [73, 74].

##### Hypothalamus–pituitary–adrenal axis

Some trials have shown that immediate postoperative morning cortisol level is a reliable marker of HPA axis function and accurately predicts postoperative secondary adrenal insufficiency. Marko et al. [84] studied 100 patients undergoing pituitary surgery and found that postoperative cortisol level ≥ 15 µg/dL (≥ 417 nmol/L using an immunoassay) was a sensitive and accurate predictor of normal postoperative HPA axis function, with a positive predictive value of 99%. In agreement, Auchus et al. [58] examined pituitary function in 28 NFPA patients before and after TSS, finding that morning cortisol level is a reliable marker of HPA axis function and provocative testing should be reserved for selected patients. In case of diagnostic doubts, serial morning cortisol evaluation seems to be useful [58, 85, 86]. Ambrosi et al. [87] has suggested that low serum dehydroepiandrosterone sulfate is a more reliable marker than basal morning cortisol for the assessment of HPA function [87] but this is rarely used in clinical praxis.

The insulin tolerance test (ITT) is considered the gold standard among provocative tests, since it evaluates the integrity of the whole HPA axis. However, ITT may have serious side effects and it is contraindicated in older patients and in patients with comorbidities such as epilepsy and ischemic heart disease [88].

The high-dose (250 µg) short Synacthen test (SST) is widely used to test HPA axis function. Adrenocorticotropic hormone deficiency gradually leads to adrenal atrophy, but the length of time over which this happens remains unclear. Concerns have therefore been raised on the reliability of SST immediately after pituitary surgery because there may be a normal response to SST despite having secondary adrenal insufficiency [89]. Furthermore, some studies have reported that HPA axis dysfunction in the early postoperative period may normalize 1–3 months after surgery, suggesting that neither SST nor ITT is helpful immediately after surgery and patients should be tested later [90, 91]. Some studies suggest that low-dose (1 µg) SST is more concordant with ITT than the high-dose (250 µg) SST in the early postoperative period [90], while other studies do not support this finding [89, 92].

Hydrocortisone is the most commonly used glucocorticoid replacement in patients with confirmed secondary adrenal insufficiency. A typical starting dose consists of 10–12.5 mg/day, which is then titrated based on clinical features. In patients with partial adrenal insufficiency, the use of conventional replacement doses may lead to excessive GC exposure and should be avoided. Whether the optimal management of partial adrenal insufficiency is to use lower doses (hydrocortisone 5–10 mg) or only use stress doses when needed is unclear [93].

Munro et al. [94] reported that approximately one in six patients with secondary adrenal insufficiency recover adrenal function, even up to 5 years after surgery [94]. Regular re-evaluations should therefore be performed, at least during the first 6–12 months postoperatively, by using morning serum cortisol before first morning dose and provocative tests when needed to prevent unnecessary GC replacement therapy.

##### Hypothalamus–pituitary–thyroid axis

The frequency of central hypothyroidism in NFPA patients varies from 18 to 43% preoperatively, and 16–57% postoperatively [31, 47, 48]. The diagnosis of central hypothyroidism is mainly biochemical, based on finding a low serum free thyroxine (FT4) concentration in combination with inappropriately low, normal, or only mildly elevated serum thyrotropin (TSH) concentration [95]. Neither serum triiodothyronine (FT3) level nor the thyrotropin-releasing hormone (TRH) test is considered a reliable test of central hypothyroidism [95–97]. The diagnosis is further complicated by the fact that some patients with low-normal FT4 concentration may have mild central hypothyroidism [95]. In these patients, FT4 concentrations should be followed and thyroxine replacement initiated if FT4 level decreases by 20% or if symptoms develop [98]. In addition, it is important to keep in mind that GH-deficient patients with low normal FT4 have increased risk of developing central hypothyroidism after GH therapy has been initiated. These patients should receive thyroxine if serum FT4 level decreases below the reference range [99].

##### Hypothalamus-pituitary–gonadal axis

Hypogonadotropic hypogonadism is reported in half of men with NFPAs preoperatively. Pituitary surgery restores normal total serum testosterone (T) concentrations in 71% of cases [100]. The presence of low total T, with low gonadotropin concentrations on two occasions is indicative of central hypogonadism [101]. If the diagnosis is doubtful, assessment of sex hormone-binding globulin and free T should be performed [101].

Premenopausal women with hypogonadotropic hypogonadism frequently present with menstrual irregularities, amenorrhea, impaired ovulation, and infertility. Low serum estradiol levels with non-raised gonadotropin levels are needed for diagnosis [56]. Preoperatively, 25% of women with NFPAs have hypogonadism [102]. In 15% of women with NFPA, hypogonadism improves following pituitary surgery [102].

##### Somatotropic axis

GH deficiency (GHD) is described in 79% of NFPA patients in the early postoperative period [103]. Recovery of the somatotropic axis function has been reported within 1–2 years after surgery and this occurs more commonly in younger patients and in patients with isolated GHD [103].

It is important to note that provocative testing of the somatotropic axis should be performed only after other hormone deficiencies have been adequately replaced. Therefore, testing of the somatotropic axis sooner than 6–12 months after surgery is not recommended.

Insulin growth factor-1 (IGF-1) levels are not reliable for assessment of GHD, as 20% of patients with GHD have normal IGF-1 levels [104]. Instead, patients with suspected GHD should be evaluated with a provocative test [105]. The ITT test is considered the gold standard and it allows to assess both the somatotropic axis and the HPA axis. The growth hormone-releasing hormone-arginine test is generally well tolerated and has therefore gained wider use [104–106]. In addition, recent studies have showed that macimorelin, an orally active GH secretagogue receptor agonist, is an accurate and safe diagnostic test for GHD diagnosis compared to ITT [107, 108]. In patients with three other pituitary hormone deficits, together with a low IGF-1, a stimulation test for GHD is not needed [56].

#### Postoperative radiological assessment

Direct postoperative MRI can be misleading due to debris, blood, and packing material following the surgical procedure. Therefore, MRI is usually performed 3–6 months after surgery, when most of the postoperative changes have disappeared [7, 52, 109, 110]. According to recent studies, early MRI has nowadays significantly higher sensitivity and specificity for detecting residual tumor than previously reported, providing valuable information to guide future care [111, 112]. The intervals for further radiological follow-up should be decided based on individual characteristics such as residual tumor size and distance from the optic chiasm.

#### Postoperative ophthalmologic assessment

In patients with decreased visual acuity preoperatively, postoperative overall improvement is recorded in 68% of cases, whilst 5% deteriorate [20]. Patients with visual field deficit have better prognosis, with an overall improvement in 81%, a complete recovery in 40%, and a deterioration in only 2% [20]. Longer duration of visual field deficits as well as severity of visual symptoms have been associated with worse postoperative visual outcomes [113–116].

Visual defects improve progressively after surgical treatment for NFPAs, especially during the first postoperative year [117]. It has been suggested that visual examination should be performed 3 months after surgery, then every 4–6 months until visual function stabilizes [42]. Annual assessment may then be performed and individualized depending on the visual status and the size and distribution of any tumor remnant [42].

### Long-term aspects of management

Patients with NFPAs have a lower chance of remission than patients with functioning pituitary adenomas [118]. NFPAs may progress after surgical treatment, with regrowth rates of 15–66% in NFPA patients treated with surgery alone and 2–28% in those treated with surgery and radiotherapy [119, 120]. Therefore, long-term radiologic surveillance after treatment of NFPAs is recommended. Recurrence rate of NFPAs peaks between 1 and 5 years after surgery and decreases after 10 years [118]. Therefore, 10 or more years of postoperative imaging is indicated, with some studies suggesting a lifelong monitoring, in particular in patients with tumor remnants [119–121].

No convincing prognostic factors for NFPA recurrence have been found so far. Roelfsema et al. [118] have showed that clinical factors such as age, sex, tumor size, and tumor invasion have limited predictive value for tumor progression. On the other hand, Ki-67 has been described as an independent cellular marker of tumor progression and recurrence [122, 123]. Recently, Raverot et al. [124] have suggested a classification of pituitary tumors into five grades that can be used by clinicians to predict tumor behavior postoperatively. This grading system is based on predictor factors, such as tumor invasion on MRI, immunohistochemical profile, mitotic index, Ki-67, and p53 positivity that can be used to identify patients with high risk of tumor recurrence or progression [124].

According to the recent WHO classification, silent corticotroph tumors (e.g. approximately 15% of all NFPAs), and sparsely granulated somatotroph tumors (e.g. circa 2% of all NFPAs) are usually more aggressive since they tend to have an invasive growth and a high recurrence rate [5]. Furthermore, Lee et al. have shown that the extent of resection and adjuvant treatments are independent prognostic factors for progression-free survival [125]. In another study, combination treatment with surgery and radiotherapy were found to be more effective than surgery alone in preventing tumor recurrence [46]. However, there are concerns about long-term complications of radiotherapy (e.g. hypopituitarism, radiation-induced optic neuropathy, increased risk of cerebrovascular events and secondary brain tumors) [54, 126]. Therefore, radiotherapy is usually reserved for cases with incomplete resection with histology showing high proliferative activity and recurrence after repeated surgical procedures [45, 126]. Development of new reliable diagnostic tools that could predict tumor progression rate would be helpful to better identify patients who should be treated with radiotherapy [45].

Available data suggest that medical therapy with dopamine agonist may have a positive effect in NFPA patients with tumour remnant [127, 128]. However, the efficacy of this treatment remains controversial since no randomized controlled trials have been performed so far. Finally, chemotherapy may be considered in selected patients with aggressive adenomas after failure of standard therapies [129, 130].

Despite NFPAs being considered benign tumors, patients with NFPAs have excess morbidity and modestly increased mortality, mainly related to circulatory, respiratory, and infectious diseases [3, 9, 131]. Interestingly, a reduction in mortality among women with NFPA has been observed during the last two decades [132]. This positive development could be explained by the decreasing prevalence of hypopituitarism recorded over time, that could be an effect of improved surgical techniques [132].

## Conclusion

In this paper we have reviewed the pre-, peri- and postoperative management of patients with NFPAs. Despite being histologically benign tumors, NFPAs are associated with long-term comorbidities, impaired well-being, and reduced long-term survival. There is limited evidence of how to guide the overall management of NFPAs in relation to the surgical procedure since treatment and follow-up strategies have not been formally evaluated in prospective randomized trials. Using available published data and data from published expert statements [28, 29, 69, 70] together with our own praxis, we have suggested a structured management strategy.

Patients with NFPAs should be treated in centers of excellence for pituitary tumors [133]. Surgical treatment should be performed with a transsphenoidal approach by an expert neurosurgeon dedicated to pituitary surgery and pre- and post-operative care should be carried out by a dedicated neuroendocrinologist [133]. Careful optimization of treatment and follow-up strategies as well as a multidisciplinary approach may have a significant impact on long-term outcomes both in terms of quality of life and survival.
